# The Aging Bowel Dysfunction and Elderly Vulnerability towards COVID-19 Infection

**DOI:** 10.3390/life11020097

**Published:** 2021-01-28

**Authors:** Aaron Lerner, Mark F. McCarty

**Affiliations:** 1 The Zabludowicz Center, for Autoimmune Diseases, Sheba Medical Center, Tel-Hashomer 5262000, Israel; 2 Catalytic Longevity, San Diego, CA 92109, USA; mmccarty@catalyticlongevity.org

**Keywords:** COVID-19, gastrointestinal tract dysfunction, elderly, morbidity, nutraceuticals

## Abstract

Severe acute respiratory syndrome coronavirus 2, primarily a respiratory tract virus, also affects the enteric organs. The most affected sector of the community are the retirement and nursing home elderly residents. Along their life the senescent gastrointestinal functions are deteriorating and failing to fully execute their digestive, absorptive, mucosal barriers, and immune protective duties. Adding the decreased motility, increased intestinal permeability, dysbiosis, morbid chronic disease background, the consumed polypharmacy enteric adverse effects to the presence of the SARS-CoV-2 host receptor along the intestinal tracts put the basis for the current hypothesis. It is hypothesized that the disadvantages and failures of the aging enteric tract contribute to the elderly morbidity and mortality during the current new coronavirus pandemic. In a more optimistic look, several nutraceuticals can prevent or restore the dysfunctional intestinal barrier functions, mainly in the elderly and potentially in those who are SARS-CoV-2 infected.

## 1. Introduction

The actual coronavirus pandemic is an outbreak of the novel acute infectious respiratory disease that started at the end of 2019 in the mega-metropolis of Wuhan, a Chinese province of Hubei. The local outbreak started in China on January 2020, spread epidemically and became, quite quickly, a pandemic. The outbreak is caused by a new coronavirus, named SARS-CoV-2. This virus is also known as a novel coronavirus that induces the COVID-19 disease [1].

As for today, most of the scientific publications concluded that this SARS-CoV-2 virus has a zoonotic origin, as are most of the human viral epidemics, however, animal 0 reservoir is not yet identified [2]. The coronaviruses are an extensive zoonotic viral family, spread worldwide and span numerous animals: bats, piglets, pigs, felines (cats), horses, camels, and swine [3,4,5,6,7]. The actual theory is that we are dealing with a new mutation or a recombinant chimera inside the coronavirus [1]. It is a very contagious, aggressive disease with high disability, morbidity, and mortality. Currently, the Chinese epidemy is contained in contrast to the USA, where, as for the end of November 2020, there are more than 14,300,000 affected and around 280,000 deaths. While the upper respiratory airways and the lungs are the main target of the virus, it appears that the heart, vessels, liver, kidneys, and the intestine are affected as well [8,9]. The gastrointestinal (GI) tract is not resilient to the virus. In fact, enteric symptoms are prevalent, ranging between 8% and 49% of patients, the major ones are diarrhea, vomiting, nausea, abdominal pains [8,9,10,11,12,13]. SARS-CoV-2 cohabitates the gut and is excreted in patients’ stools [12,14,15,16,17,18,19,20,21,22]. Colonic biopsy samples positivity has been consistently documented and multiple publications argue for potential fecal–oral transmission of the disease [13,18,23,24,25,26]. One of the interesting aspects of the disease is its differential age targeting and distribution and potential infectivity. It is clear that the elderly patients are prone to be infected and have more morbid and lethal disease, compared to children [27]. Available epidemiological observations hints to the fact that COVID-19 seems to be uncommon in children [28,29]. On the contrary to children, the prognosis, morbidity, and mortality of SARS-CoV-2 infected elderly is significantly higher than young and middle-age patients [30]. Notably, patients aged 60 years and over have a significantly higher mortality rate (5.3%) than that of patients below 60 years (1.4%) [30,31]. Multiple publications expand on respiratory and lung vulnerability during the ageing process, however, the senescent GI tract performances that face the new coronavirus are scarcely reported. The present review summarizes the age dependent changes and decline of the gut functions in the elderly. The hypothesis is that the elderly enteric dysfunction put them in a higher risk for COVID-19 severe disease and compromised outcome.

## 2. Gut Dysfunctions in the Elderly

### 2.1. Digestive and Absorptive Dysfunction

The digestion and absorption of food is a multi-processed mechanism which is dependent on endogenous secretion of enzymes, exogenous microbial metabolic availability, GI motility, entero-endocrine hormonal activity, and the enteric nervous system control and regulation. During the ageing period, many physiological functions are declining including GI performances. Salivation, olfactory, gustatory, and visual food perceptions, oral and dental health, digestion and absorption and lactose intolerance, diminished appetite and water and food consumption are few of them [32,33]. Those are some of the reasons of malnutrition in older people above the age of 65 years. Their compromised nutritional status is one of the most relevant conditions that negatively affects elderly health and even can predict their preterm death [34]. Chewing activity, enteral hormone that regulates appetite, and endogenous digestive enzyme capacity deteriorate along the third stage of life. Salivary, gastric, duodenal, jejunal, ileal, and colonic enzymatic performances are reduced. On the mucosal part, the corresponding specific absorptive receptors, pathways, apical and trans-enterocytic and enter-enterocytic absorptive mechanisms are also suboptimal or reduced [32,33,34]. There is a reduced digestion of sugars, proteins, and fats. The corresponding specific enzymes, namely, the saccharidases, numerous proteases, and various lipases quantities and digestive capacities decline during ageing [33]. Bile acids pH buffering, fat emulsifying, ileal reabsorption, and the microbiome digestive capacities are as well, gradually decreasing with age [32,33,34]. On top of the above the decrease in the surface area of the small intestine following the ageing degeneration of the villi may lead to blunted absorption of food components [35].

### 2.2. Failure of Mucosal Barriers and Immune Dysfunction 

The largest immune system resides in the enteric mucosa, facing uncountable foreign factors, exogenous antigens, toxins, allergens, carcinogens, microbes, viruses, parasites, and other detrimental environmental factors. The largest number of lymphocytes reside in the GI mucosa, represented by the gut-associated lymphoid tissue (GALT). A recent study gives us an overview of candidates from genes and pathways regulated in aging and age-related diseases. Using gene expression databases, the authors identified 44 markers in seven categories (inflammation, mitochondria and apoptosis, calcium homeostasis, fibrosis, neuromuscular junction and neurons, cytoskeleton and hormones, and other) with potential and priority as frailty biomarkers related to aging and age-related diseases [36]. The following markers strongly related to cellular senescence, many involved in GI functions, were IL-6, CXCL10, sVCAM/sICAM, regucalcin, calreticulin, TGFβ, PAI-1, HMGB1, α klotho, FGF23, IGF-1, and miRNAs panel. Those senescent markers need to be experimentally explored in animal models and in the human elderly GI organs for validation and assessing their prognostic, diagnostic, prognostic, and therapeutic potentials.

The following mucosal barriers are weakened during senescence: 

Alpha and β defensins, antimicrobial and cytotoxic peptides involved in host defense and in immunomodulation, expressed in alimentary track, particularly in Paneth cells and epithelial cells [33,36]. Mucus layers, which covers the epithelium is a prime barrier that keeps dysbiota and pathobiota in the luminal bay. In *Helicobacter pylori* positive elderly, the thickness of the mucus layer is reduced [37] and the adhesive ability of *bifidobacteria* to attach to the mucosa of elderly is declined [38].

As for the frailty and dysfunction of the human senescent mucosal immune systems, various immune functions are failing to execute their pivotal protective function. The local immunosenescence is expressed by reduced secretion of specific immunoglobulin A [39], mature M cells density in the Peyer’s patches of aged mice is decreased [40], tolerance to naïve antigens is declined [41], Peyer’s M enterocyte transcytosis of luminal antigens is weakened [40], dendritic cells density and functionality is decreased, thus, compromising presentation of processed antigens to the mature T and B lymphocytes [42]. All those integrated mucosal immune responses against pathogens, generated in the GALT, decline along the ageing process [33,35,43,44].

Facing the thinner mucosal layer, the reduced immunocompetent cells volume and the surge in proinflammatory cytokines (IL-1b, IL-6, TNFα), it can be summarized that the physical and chemical barriers, the reactive and innate mucosal immunity are compromised in the aged gut mucosa [33,43,44,45].

### 2.3. Decreased Microbiome/Dysbiome Ratio

The studies on microbiome/dysbiome ratio and microbial composition and diversity are complicated since the enteric microbiome is influenced by lifestyle, age, nutrition, drugs, associated chronic diseases, community versus long-term residential care dwellers, and many other environmental factors. Those parameters impose scientific burden and bias when interpreting the results. The impact of aging on the enteric microbiome is not an exception and explains the variability in the results [46,47,48,49]. Most studies reinforce the decreased stability and increased diversity of the intestinal microbiota during aging. Some reported *bifidobacteria*, a putatively protective lactic acid producing bacteria, and anaerobes, to be under-represented in the flora of advance age [47,50]. Some observe increased [47,48,50] and some decreased [48,49] diversity, but the studied elderly are extensively different and are not comparable. Enhanced colonization of pathobionts, like *Staphylococcaceae*, *Enterobacteriaceae*, and *Enterococcaceae*, paralleled by a decline in beneficial bacteria such as short-chain fatty acid-producing microbes [51], were reported [49]. Some suggest that the reduction in the putatively protective bifidobacterial and the reduced production of short chain fatty acids increase disease risk in elderly people [52]. The questions of cause, consequence, or coevolution, are, as yet, unresolved [48], as is in other GI chronic conditions like celiac [53] or Crohn’s diseases [54]. Hopefully, future identification of specific proteinome or metabolome or metabolic mechanisms that are driven by the gut bacteria in the aging intestine will clarify those questions [55].

### 2.4. Decreased Motility

The motility and segmental transit times during aging were extensively reviewed, but the conclusions are quite controversial [33,35,56]. Despite the heterogeneity of the studies, several trends can be outlined. Symptomatically, the elderly is suffering from higher threshold for tastes, swallowing difficulties, nausea, dysphagia, heart burning due to gastro-esophageal reflux disease, constipation, and fecal incontinence. The etiology of constipation can be due directly to advance age; however, a plethora of other factors can contribute. Low fiber and fluid intake, side effects of drugs, associated morbid diseases, and reduced physical activity, are some of them. Interesting are the observations of age-dependent degenerative neural mechanisms in the myenteric plexus of the GI nervous system that can impact not only motility but also appetite, food intake, digestion, absorption, local and central gut performance regulation [33,35,56,57].

### 2.5. Increased Intestinal Permeability

Intestinal permeability is tightly regulated and evolutionary conserved, in order to keep foreign antigens inside the enteric lumen [58,59,60]. Compromised tight junction functional integrity resulting in increased enteric permeability was documented in elderly people [33,35,45,61,62,63]. Adding the failure of mucosal barriers and immune dysfunction, the dysbalanced microbiota to the digestive and absorptive abnormalities of the senescent human gut further potentiate the enhanced gut permeability.

### 2.6. Increased Chronic Diseases That Induce Gut Dysfunction

The list of associated chronic diseases that contribute to the COVID-19 morbidity and mortality is extending. Hypertension, diabetes, cardiovascular disease, chronic lung disease, chronic kidney disease, cancer, immune dysfunction states, and heavy smoking, are on the list [64,65]. Those conditions are prevalent in >65 years old people and being a male put them at a greater risk. It should be stressed that all those associated conditions affect GI functions and performances and are adding to the GI vulnerability of the current SARS-CoV-2 pandemic.

### 2.7. Increased Nutritional Deficiencies

Balanced personal diet and sufficient caloric intake are crucial for good health status, during the aging period since aging and sickness share paths. Due to many reasons cited above, the nutritional status of the elderly put them at risk for nutritional deficiencies. It is not only the prevalent aging associated malnutrition; hence, specific nutritional factors’ deficiencies were described. Iron, B12, B1, B6, folate, vitamin D, zinc, and selenium are the most important ones [66,67]. It goes without saying that those deficiencies and the associated malnutrition, impair normal elderly GI functions and enhance age-dependent peripheral organs pathologies, mainly in the cardiovascular, musculoskeletal, peripheral and central nervous, immune and skin systems [33,68].

### 2.8. Increased Drug Consumption with Gastrointestinal Side Effects

Along aging, drug consumption increases and puts the elderly at risk for adverse drug reactions. The GI tract side effects are one of the most often reported [69]. NSAIDs and/or aspirin and antibiotics are on the top of the list with their upper and lower bowels’ complications. On the other hand, the frequently prescribed polypharmacy brings further morbidity to those vulnerable aged patients [70]. Many advanced age GI symptoms and diseases are pharmacologically treated. Gastro-esophageal reflux, gastroparesis, constipation, diarrhea, peptic ulcer disease, *Helicobacter pylori* gastritis, irritable bowel syndrome, IBD, chronic renal and liver disease, osteopenia/osteoporosis, and the above cited nutritional deficiencies, are on this list. All those facts further jeopardize the elderly GI functional integrity. Table 1 summarizes the various aspects of the GI dysfunctions during the aging period of life.

## 3. The Aging-Gut–SARS-CoV-2 Interrelationship

Since December 2019, a newly identified coronavirus is spreading worldwide, affecting almost all the countries on the globe. As of December 2020, there are around 64,000,000 infected and 1,500,000 deaths. It originated from Wuhan, in Hubei province in China but concentrated in several hot spots like USA, Italy, Spain, Iran, India, and Russia. The SARS-CoV-2 targets advanced age people, many of them with associated chronic diseases, residing in home-care centers [27,28,29,30,64,65]. In Israel for example, out of 1710 deaths, 619 came from nursing homes for the elderly. Normally, they represent 1% of the general population, however, their COVID-19 death rate approaches 36%. No doubt that the respiratory tract and the lungs are the most affected organs, but screening the literature, coming earlier from China and latter from other affected countries, the GI tract is also targeted by the SARS-CoV-2 virus. As mentioned above, multiple observations and facts argue for the gut being a target organ. GI symptoms [8,9,10,11,12,13], dwelling in the gut lumen and excreted in stool [14,15,16,17,18,19,20,21,22], induced GI pathology, the entire digestive tract is affected [13,15,71], stools as a way of diagnosis [20], potential fecal–oral transmission [13,18,23,24,25,26] and the observation that diarrhea is associated with prolonged symptoms and substantial viral carriage of the virus [16], strengthen the tight relation between the SARS-CoV-2 and the human intestinal tract. More so, the new coronavirus host cell receptor is angiotensin converting enzyme 2 (ACE2). This receptor exists on the esophageal lining epithelium, on the small bowel enterocytes and the large bowel colonocytes [23,72]. Adding all the above SARS-CoV-2–gut interrelationships to the fact that the most vulnerable people are the elderly with their dysfunctional GI tract, it is hypothesized that the gastrointestinal functional senescence and frailty contribute substantially to COVID-19 elderly affected mortality. The digestive and absorptive dysfunction [32,33,34] leading to malnutrition [33] and nutritional deficiencies [33,34,66,67,68], failure of mucosal barriers and relative immune dysfunction [39,40,41,42,43,44], compromised motility [33,35,56,57], and enhanced permeability [33,35,45,61,62,63], on the background of high chronic disease rate [64,65] and polypharmacy adverse effects [69,70], all impair intestinal functions. The fact that elimination of SARS-CoV-2 from stool takes longer than from the nose and throat represents a red warning [16]. It seems that the fecal–oral transmission is under investigated and might play a major role in the nursing and retirement homes of the GI vulnerable elderly. Nevertheless, the human-to-human transmission within families is occurring; in China the SARS-CoV-2 RNA has been detected in patients’ feces by PCR analysis [73].

Finally, the respiratory tract is the primary target organ of the COVID-19 disease and the main mortal factor, but, the disadvantages and failures of the aging enteric tract might contribute to their morbidity and mortality during the current pandemic. In this regard, Table 2 summarizes the GI dysfunctions that are associated with or induced by the COVID-19 agent. Comparing Table 1 to Table 2, one can see the similarity between the GI dysfunctions that are attributed to the aging process and those associated with SARC-Cov-2 infections. Interestingly, in both, digestion, absorption, mucosal barriers and immune dysfunctions, macrobiomic homeostasis, motility and gut permeability, and nutritional health are compromised [74,75,76,77,78,79,80]. More so, COVID-19–gut–aging relationships are further strengthened by the following observations. 1. The GI dysfunctions and the intestinal symptoms of the elderly might increase the severity and duration of COVID-19 infection [81]. 2. ACE2 expression is increased during aging [82,83], thus potentiating the elderly’s vulnerability to infection. 3. Many older people have comorbidities and associated chronic diseases, including cardiac, renal diseases, and hypertension, thus chronically consuming angiotensin 1 receptor blockers. Recently, this drug-induced over expression of ACE2 was suggested for the elderly subjects’ enhanced susceptibility to severe COVID-19 [84]. Taken together, the senescent gut cross talks with the SARS-Cov-2 virus are suggested to increase the susceptibility of the elderly to COVID-19 disease. Unfortunately, efficacious pharmacological therapy for intestinal barrier dysfunction during aging is lacking; hence, strategies for nutraceutical support of barrier function are of significant interest.

## 4. Potential Nutraceutical Support of Intestinal Barrier Function

Intestinal enterocytes are linked by apical tight junctions that are highly conserved during eukaryotes evolution including humankind. Their pivotal function is to limit the paracellular absorption of foreign proteins/peptides, bacterial components and toxins, various allergens and carcinogens, thus preventing acute and chronic diseases. In addition, they serve to prevent massive fluid and electrolyte losses and ensuing metabolic abnormalities. Perturbated homeostatic function of this barrier can increase risk for infections, systemic and hepatic inflammation, food allergies, cancers, and neurodegenerative and autoimmune disorders [58,59,60]. Taking into consideration the gastrointestinal dysfunctions in the elderly and those associated with SARS-Cov-2 infection described above (table 1+2) and the present hypothesis relaying SARS-CoV-2 susceptibility to the senescent gastrointestinal tract, a major question arises: can nutraceuticals prevent or reverse enteric dysfunction in the elderly? Nutraceuticals are nonpharmaceutical alternatives to drugs which may exert physiological benefits. Any product derived from natural food sources that can provide health benefits in addition to the basic nutritional support provided by foods, can be defined as a nutraceutical. In this regard, several of those nutraceuticals have the potential to support effective intestinal barrier function in the elderly.

Various mechanisms and pathways support the formation, maintenance, and functions of the enter-enterocyte’s tight junction zipper-like complex. Glucagon-like peptide-2 (GLP-2) is produced in response to various signals by special neuroendocrine L cells in the intestinal mucosa [85]. This peptide acts locally on subepithelial fibroblasts to stimulate their secretion of insulin-like receptor I (IGF-I), which in turn acts to boost PI3K/Akt/mTORC1 signaling in enterocytes. This exerts an anabolic effect, promoting proliferation and suppressing apoptosis of enterocytes, but also supporting the formation and maintenance of tight junctions [85,86,87]. Notably, the PI3K/Akt/mTORC1 signaling pathway triggered by IGF-I activity on enterocytes has been shown to upregulate mRNA and protein levels of a range of proteins required for tight junction formation and function, including occludin, claudins, and ZO-1 [88]. Hence, nutraceuticals which promote GLP-2 secretion from L cells can support intestinal barrier function; these include effective pre/probiotics, glycine, and glutamine [89,90,91,92,93]. Moreover, diets rich in soluble fiber or resistant starch, by inducing bacterial production of short-chain fatty acids that act as secretagogues for L cells, can likewise support intestinal barrier function [51,88,89,90,91,94].

The favorable impact of AMPK activation on enteric barrier functions is well documented. It is mediated, at least in part, by increased expression of caudal type homeobox 2 (Cdx2), a master transcription factor driving differentiation of gut enterocytes [95]. Hence, pharmaceutical or nutraceutical AMPK activators, such as metformin, berberine, and the short chain fatty acids produced by healthy microflora, can be beneficial to intestinal barrier function. The nutraceutical berberine, a compound found in various Chinese medicinal herbs, is widely employed for diabetes management in China [96,97]. Likely as a consequence of its ability to stimulate AMPK activity, berberine is reported to have favorable effects on the intestinal barrier and tight junction maintenance [96,97,98].

Another factor favorable to the intestinal barrier is the beta-isoform of the estrogen receptor (ERβ); agonists for ERβ aid barrier function, in part, by inducing increased expression of occluding [99]. Fortuitously, when feasible levels of soy products are ingested, the plasma levels of unbound, unconjugated genistein and S-equol that result are sufficient to achieve significant activation of ERbeta, with minimal impact on ERα activity (which is why soy does not induce feminizing effects or increase risk for estrogen-dependent cancers) [100,101,102].

Inflammation of intestinal enterocytes, associated with oxidative stress, is more common during aging [35]. Oxidant stress impedes tight junction formation via activation of the MAP kinases, JNK, p38, and ERK1/2, which oppose the assembly of tight junctions. Appropriate antioxidants have the potential to suppress activation of these kinases by inhibiting oxidant production, promoting catabolism of hydrogen peroxide, or reversing the sulfhydryl-oxidizing effects of hydrogen peroxide on signaling proteins—and hence, have potential for supporting enteric barrier function in the context of inflammation. Antioxidants which may be useful in this regard include phycocyanobilin (a biliverdin metabolite which functions as a prominent light-harvesting chromophore in cyanobacteria such as spirulina, and which can inhibit certain isoforms of NADPH oxidase); phase 2 inducer nutraceuticals such as lipoic or ferulic acids; N-acetylcysteine, a source of the L-cysteine that is rate-limiting for synthesis of the prominent intracellular antioxidant glutathione [103,104,105,106,107,108]. Table 3 summarizes the various nutraceuticals, the mediators involved, and their impact on tight junction performance. Those considerations suggest that rationally designed functional foods or complex supplementation programs may have clinical potential for supporting and restoring healthful intestinal barrier function during aging.

## 5. Conclusions

The most affected sector in the current COVID-19 pandemic is the nursing and retirement homes elderly. Most of the clinical and scientific interests has been referred to the respiratory tract, but the digestive ways are also affected. Along human life, gut performances are weakened, and GI dysfunctions develop to the detriment of the elders. SARS-CoV-2 gut receptors close to the viral enteric presence, enteric pathology, and viral stool excretion, on top of the elderly local vulnerability is the basis for the present hypothesis of gastrointestinal functional senescence contribution to the COVID-19 elderly affected mortality. In this regard, several nutraceuticals can prevent or restore intestinal barrier dysfunctions by supporting tight junction functional integrity.

## Figures and Tables

**Table 1:** A summary of the gastrointestinal dysfunctions in the elderly.

Category of Gastrointestinal Dysfunction	Increase/Decrease	Reference
Digestive	↓	[32,33,34]
Absorptive	↓	[32,33,34,35]
Mucosal barriers	↓	[33,36,37,38]
Immune	↓	[39,40,41,42,43,44]
Microbiome/dysbiome ratio	↓	[46,47,48,49,50,51,52]
Motility	↓	[33,35,56,57]
Permeability	↑	[33,35,45,61,62,63]
Associated chronic diseases	↑	[64,65]
Nutritional deficiencies	↑	[33,66,67,68]
Drug consumption with gastrointestinal side effects	↑	[69,70]

**Table 2:** Intestinal dysfunctions in COVID-19 infections.

Gastrointestinal Dysfunction in COVID-19	Increase/Decrease	Reference
Digestive	↓	[74]
Absorptive	↓	[74]
Mucosal barriers	↓	[75]
Immune functions	↓	[76]
Microbiome/dysbiome ratio	↓	[77]
Motility	↓↑	[78]
Permeability	↑	[79]
Nutritional deficiencies	↑	[80]

**Table 3:** A summary of various nutraceuticals, the mediators involved, and their impact on tight junction’s performance.

Nutraceuticals	Intermediates	Tight Junctions’ Performance	References
**Prebiotics/probiotics**	Glycine, glutamine, butyrate, glucagon-like peptide-2, insulin-like growth factor-I, PI3K/Akt/mTORC1	Improved	[51,77,78,79,80,83]
**Prebiotics/probiotics**	Butyrate, AMPK	Improved	[51,77,78,79,80,83]
**Berberine**	AMPK	Improved	[85,86,87]
**Soy isoflavone**	ER-beta	Improved	[88,89,90,91]
**N-acetylcysteine**	NADPH oxidase, JNK, p38, ERK1/2	Suppressed	[96]
**Nrf2**	NADPH oxidase, JNK, p38, ERK1/2, HO-1	Suppressed	[92,93,94,95]
**HO-1**	NADPH oxidase, JNK, p38, ERK1/2	Suppressed	[92,93,94,95]
**Phycocyanobilin**	NADPH oxidase, JNK, p38, ERK1/2	Suppressed	[92]

## Abbreviations

Angiotensin converting enzyme 2—ACE2; gastrointestinal—GI; glucagon-like peptide-2—GLP-2; caudal type homeobox 2—Cdx2; estrogen receptor-beta—Erβ; insulin-like receptor I—IGF-I.
